# XDR-tuberculosis in HIV infected late presenter patient – Case report

**DOI:** 10.1186/1471-2334-14-S7-P79

**Published:** 2014-10-15

**Authors:** Marina Alina Lungu, Augustin Cupsa, Florentina Dumitrescu, Andreea Cristina Stoian, Mihai Jianu, Victor Grecu

**Affiliations:** 1 “Victor Babeş” Clinical Hospital of Infectious Diseases and Pneumology, Craiova, Romania

## Background

Tuberculosis (TB) is one of the most frequent diseases met in HIV infected patient, co-infection HIV-TB being an important cause of morbidity mortality in late presenter patients.

## Case report

We report the case of a 31 years old female, who was hospitalized in April 2011 in “Victor Babeş” Hospital of Craiova for fever, wet cough, weight loss, symptoms that had been evolving for 2 months. The clinical exam shows: febrile patient, BMI=20.3 kg/sqm, pallor, generalized lymphadenopathy, oral candidiasis, crackles bilaterally, tachypnea, chest wall retraction, patient’s O2 saturation=90%. Laboratory tests: L=7,200 cells/cmm (11% un-segmented neutrophils, 66% segmented neutrophils, 14% lymphocytes), ESR=135/140 mm. Chest X-ray: micronodular opacity in both pulmonary area, medium intensity opacity apical and retroclavicular on left side; BK-smear was negative.

Because the patient had a history of repeated bacterial pneumonia, it raised suspicion of HIV infection, which it was confirmed (ELISA antibody anti HIV 1,2 positive + Western blot positive), the patient being a late presenter with severe immunosuppression (CD4=23 cells/cmm) and high viral load (VL=533,646 copies/mL). After two weeks of treatment with antibiotics, it was initiated antiretroviral treatment (ART): AZT/3TC+ EFV well tolerated.

In May 2011 the patient was diagnosed with secondary infiltrative-nodular TB of right superior pulmonary lobe (repeated BK negative smears) and it was initiated standard TB treatment. In July 2011 because of unfavorable clinic evolution thoracic CT exam was performed that showed an opacity of inferior lobe of dorsal segment and mediastinal lymphadenopathy and it was repeated the BK smear-negative; it was changed the TB terapy-H300 R600 Z2000 E 1600 ciprofloxacin 1 g/day 7/7 and in the same time patient had received meropenem 3g/day for 21 days.

The culture for *Mycobacterium tuberculosis* became positive and antibiotic test resistance showed XDR-TB (resistance to rifampin, isoniazid, ethambutol and amikacin). Between November 2011 and May 2012 the patient followed individualized TB therapy: cycloserine+pyrazinamide+prothionamide+streptomycin, then the same combination except streptomycin for next 12 months. The patient had a very good adherence to TB and antiretroviral therapy, with excellent clinical and immunovirological evolution: May 2012 CD4 was 193 cells/cmm and undetectable VL-HIV; the chest X-ray showed left subclaviculary and left parahilar fibrosis.

## Conclusion

A late presenter HIV infected patient with XDR-TB had a favorable outcome because of individualized TB therapy (possible because of repeated search for etiological diagnostic) and probably because of treatment with meropenem, but mostly because of good adherence to TB and antiretroviral therapy.

## Consent

Written informed consent was obtained from the patient for publication of this Case report and any accompanying images. A copy of the written consent is available for review by the Editor of this journal.

